# Exhibiting Good Health: Public Health Exhibitions in London, 1948–71

**DOI:** 10.1017/mdh.2017.72

**Published:** 2018-01

**Authors:** Alex Mold

**Affiliations:** Centre for History in Public Health, London School of Hygiene and Tropical Medicine, 15-17 Tavistock Place, London, WC1H 9SH, UK

**Keywords:** Public health, Exhibitions, Medical officers of health, London

## Abstract

This article examines the changing nature of public health services and their relationship with the public in post-war Britain by an analysis of the exhibitions mounted by Medical Officers of Health (MOsH) in London. Focusing on the period 1948–71, the article explores a time when public health practice, and the problems it faced, were in flux. A decline in infectious disease and an increase in chronic conditions linked to lifestyle required a new role for public health services. Exhibitions were one of several methods that MOsH used to inform the public about dangers to their health, but also to persuade them to change their behaviour. The exhibition, though, offers a unique insight into the relationship between public health authorities and the public, as exhibitions brought MOsH into direct contact with people. It is suggested that in the MOsH exhibitions we can find signs of a new relationship between public health practitioners and the public. Whilst elements of the pre-war, often moralistic ideology of public health services could still be detected, there is also evidence of a more nuanced, responsive dynamic between practitioners and the people. By the end of the 1960s, ‘the public’ was increasingly being thought of as a collection of ‘publics’, including individuals, target groups and vocal respondents.

In the autumn of 1949, a reporter from the BBC attended an exhibition on ‘Clean Food’ organised by the Medical Officer of Health for the London Borough of Ealing. The journalist told the radio programme, Woman’s Hour, that ‘This business of dirty food isn’t a pleasant subject and the Public Health Committee of Ealing, in putting on this exhibition, haven’t at all gone out of their way to disguise its unpleasantness.’ The exhibition contained a model of a grocers’ shop, complete with a waxwork female assistant wearing a ‘grimy overall’ with ‘greasy, tousled hair’ and ‘one finger is wrapped in a dirty bandage’. Elsewhere in the shop, there were ‘uncovered sausages and pies and other foodstuffs’ and, more shockingly still, in between some open sacks of groceries, a large rat. The journalist noted that ‘It’s a stuffed rat, as it happens, but just in case you think that’s a sign of squeamishness – there’s a glass case around the corner showing a section of a sewer, and inside that is a real live rat. Then there are live mice and dead rabbits and doomed cockroaches and things – all there not for their horrific value but to show the public how and by what agents food can become poisoned.’ Indeed, the exhibition made a favourable impression on the reporter, and they were sympathetic towards the organisers’ view that ‘there’s no longer any excuse for the poor food hygiene which wartime difficulties made unavoidable’. The exhibition, the journalist suggested, contained ‘cleverly devised lessons’ and ‘dozens of simple, uncostly ways by which the standard could be raised’.^1^

The exhibition in Ealing might have been unusual in generating national media coverage, but it was not atypical as a method of health education in Britain during the post-war period. Exhibitions were one of several techniques drawn upon by Medical Officers of Health (MOsH) to communicate health education messages to the public. Posters, leaflets, film showings and talks were all utilised by MOsH to varying degrees. What was special about exhibitions was their use of range of different types of visual media, something contemporaries thought to be particularly effective. In 1959 the journal *Health Education* devoted a special issue to the subject of health exhibitions. Sidney Chave, a senior lecturer at the London School of Hygiene and Tropical Medicine, noted that ‘There is no doubt that the step by step simplification which can be achieved by means of a well-designed visual display can provide an effective medium for teaching, particularly where a relatively complicated subject has to be “got across”.’^2^ Moreover, F. St.D Rowntree, a health education officer at Essex County Council asserted in the same issue that the exhibition had an advantage over other means of health education. He stated that ‘impersonal methods’ of communication such as the poster or the leaflet presented ‘little opportunity for human contact or the discussion of ideas and in the main provided a means of supplying, but not necessarily imparting, factual information. These methods are of little help in producing desired attitude changes.’ Exhibitions, on the other hand, allowed the public to interact with material, something which would produce ‘an added feeling of realism; interest is stimulated and there is a greater personal involvement in the theme displayed’.^3^

For the historian, the exhibition offers something else: an opportunity to observe the relationship between public health services and the public. Exhibitions brought public health officials and the people that they served into direct contact in a way that other health education methods did not. This is important, because the post-war period was a time when the relationship between public health services and the public came to matter as never before. Towards the end of the nineteenth century, infectious disease declined as a major cause of morbidity and mortality to be replaced with chronic conditions like coronary heart disease and cancer.^4^ In the middle of the twentieth century, these conditions became linked to so-called ‘ways of living’, to behaviours like smoking that increased an individual’s risk of becoming ill.^5^ To prevent such conditions, public health practitioners needed to appeal to individuals to change their lifestyles. Health education directed towards behaviour change thus became more important. Public health practice had long included health education, but in the post-war period it became one of the chief weapons against ill-health, in contrast to previous eras where environmental and technological changes played a greater role. Getting people to alter their behaviour appeared to require a new relationship between public health services and the public, a relationship orientated not so around much what public health authorities could do for the public, but around what the public could do for itself.

The implications of such a shift were profound for both the public and public health practice. Book-ended by the establishment of the National Health Service (NHS) in 1948 and the demise of the MOsH following the reorganisation of the health service and the removal of public health from local government in 1974, this article explores a time when public health practice shifted from fighting the foes of the interwar period in matters concerning personal hygiene and motherhood, to facing the new threats to population health posed by lifestyle-related non-communicable disease. Making use of the digitised London Medical Officers’ of Health reports, the article examines the changing nature of public health services and their relationship with the public by an analysis of the exhibitions mounted by MOsH. It will suggest that public health practice was beginning to take on its new enemy, as exhibitions focused on infection and the environment, such as the issue of food hygiene, slowly gave way to those concentrating on non-communicable threats to health like safety in the home and cigarette smoking. Evidence of such activity contrasts with the picture of the post-war MOsH painted by some historians. Both Jane Lewis and Charles Webster have argued that the MOsH were slow to adapt to the changing nature of public health problems, pointing to their inability to engage with health education as a prominent sign of the MOH’s failings.^6^ Yet, other work has shown that some MOsH were able to adapt to their new circumstances, a finding that this article builds on and expands.^7^ More broadly, it is argued that in the MOsH exhibitions we can find signs of a new relationship between public health services and the public. Whilst elements of the pre-war, often moralistic ideology of public health services could still be detected, there is also evidence of a more nuanced, responsive dynamic between practitioners and a collection of ‘publics’.

The nature of post-war public health policy and practice in Britain is starting to be explored in greater depth by historians. The article begins by considering this work, as well as looking back to public health during the interwar period to gain a sense of what might have changed in the latter half of the twentieth century in relation to both health education and the practice of public health at the local level. It is suggested that there were continuities between the interwar and post-war decades, but there were also differences, as MOsH and their health education techniques were adapted to deal with new challenges. In the second section, the article will consider the content of the exhibitions organised by London MOsH. In the late 1940s and early 1950s, food hygiene was a dominant concern. But, by the mid-1950s and early 1960s, other issues, like air pollution, safety in the home and cigarette smoking, became more prominent. This would suggest that MOsH were placing less emphasis on their infection control work and moving towards persuading the public to change their behaviour to avoid chronic disease. In the third section, the article scrutinises the techniques employed by MOsH in their exhibitions. A wide range of approaches were offered, often with the explicit aim of reaching as large an audience as possible. Indeed, as discussed in the final section of the piece, MOsH exhibitions can tell us much about the relationship between public health services and the public. MOsH were keen not to preach to the converted, but to influence the behaviour of those they thought ‘most in need’ of health education. These groups consisted of many of the traditional targets of public health services: the working classes, women, and towards the end of the period, ethnic minorities. Elements of the paternalism and surveillance that had been present in the work of the MOsH in earlier decades persisted. Yet, there was also a widening of public health authorities’ gaze to include those from all socio-economic categories as well as the disadvantaged. There were a number of different ‘publics’ at work within the MOsH’s view of ‘the public’, and some were thought to be in need of more attention than others. ‘The public’, therefore, was increasingly being thought of as a collection of ‘publics’, including individuals, target groups and vocal respondents. Moreover, exhibitions offered the public a chance to ‘speak back’ to public health services. Displays were often staffed by members of the public health department, giving the public an opportunity to ask questions and raise grievances. All this points towards the development of a more dynamic relationship between the public health services and their publics, one that required greater interaction and dialogue on both sides.

## 1 Public Health and the Public in Post-War Britain

The practice of public health, and the public which it served, were both in flux in the post-war era. For many historians and other analysts, the time from the creation of the NHS in 1948 until the early 1970s was representative of a specific view of health, welfare and the citizenry. John Pickstone suggested that this was the era of ‘communitarian medicine’, of a public service built on social solidarity.^8^ The NHS was the practical realisation of T.H. Marshall’s notion of social citizenship, whereby the state provided essential social goods and services, such as medical attention, shelter and education, to be enjoyed by all citizens.^9^ As Dorothy Porter points out, social citizenship was also rooted in pre-war notions of social medicine which was concerned not only with the establishment of collective services, but also the impact of social structure on health.^10^ In the 1950s, however, she suggests that interest in social structures was replaced by a focus on social behaviour.^11^ This shift was driven in part by the epidemiological transition, and growing recognition of the impact of behaviour on health, but was also related to a renewed interest in the public and its conduct. Attempting to know the people, their attitudes and actions, was of course, nothing new, but the mid-twentieth century saw a rapid growth in social science, and the development of its methodologies, such as the survey.^12^ Social research in health, welfare, politics and in commerce proliferated.^13^ Public health practitioners and researchers also attempted to find out more about the public, what they wanted and how they behaved.

Establishing the true nature of this public, was, however, difficult. In part this was because the meaning of the public was ill-defined and unstable. ‘The public’, as the political philosopher John Coggon points out, has much in common with Benedict Anderson’s notion of imagined communities.^14^ No member of the public can know all the other members of the public, but in their minds or those of others they are part of one community. Likewise, drawing on Raymond Williams’ analysis of the ‘masses’, David Cantor notes that there was no such thing as ‘the public’, only ways of seeing it. What Cantor also suggests, however, is that the meaning of the public began to change in the post-war era. He argues that before the 1940s, the public was regarded as an undifferentiated mass. Following the establishment of the NHS, the development of consumerism and the application of epidemiological categories to the population, Cantor contends that ‘the public’ began to fragment into different groups. By the 1970s, he suggests, ‘the notion of an undifferentiated public was much harder to sustain, and differences, which might once have been portrayed as variations within the mass general public, came to be marks of different publics’.^15^

The beginnings of such fragmentation, marked by a growing focus on individual behaviour and specific population groups, can be observed in the health education work of the London MOsH, and specifically the exhibition. The digitisation of the London MOsH reports by the Wellcome Trust has helped to facilitate the analysis of public health practice at the local level from the introduction of the MOsH in the mid-nineteenth century, to their demise in the early 1970s.^16^ Each MOH was required to produce an annual report detailing the health of the population in their borough and the efforts taken to protect and improve this. The reports contain a wealth of quantitative and qualitative data, and are beginning to be made use of by historians and others.^17^ Despite their somewhat formulaic style, the reports can tell us much about the practice of public health and the relationship of the public health services with the local people. London offers a useful case-study of MOsH work, as the city was made up of a range of wealthy and less affluent boroughs and encompassed a large and diverse population. Particular problems, both real and imagined, such as air pollution and the health of immigrants, also affected the capital more than other parts of the country. This approach, however, is not without its drawbacks. Firstly, developments in London cannot be assumed to have taken place elsewhere, and the health problems faced in the capital were not necessarily the same as those encountered in other urban areas, let alone more rural ones. Secondly, the reports only cover the period up to 1971, three years before the MOsH post was formally scrapped. Thirdly, the reports detail the work of MOsH and this could lead to an over-emphasis of their role, although this is something which can be overcome by locating the MOsH within historical and historiographical context. Finally, the reports represent the views of the MOsH and their conceptualisation of the public. To get at the public’s view of public health, we need to ‘read against the grain’ of the reports, scrutinising the text for signs of resistance or reinterpretation of key messages and actions by the public.^18^

The significance of the MOsH in the twentieth century, and especially in the post-war period, has been the subject of historical debate. Lewis argued that public health practice in the twentieth century did not have an overarching philosophy, and instead let itself be defined by the activities it undertook. She was particularly critical of the work of the MOsH in the post-war era, suggesting that they lacked an understanding of their duties and role, that they were unimaginative, and failed to undertake broader epidemiological work. There were tensions between MOsH and other health professionals within the new NHS, especially GPs and, by the 1960s, social workers too.^19^ Webster was also critical of the MOsH in both the interwar and post-war periods. He argued that in the 1930s, MOsH neglected to address the impact of unemployment on health, particularly that of children and mothers.^20^ After the establishment of the NHS, Webster contends that the MOsH ignored health education on preventive issues such as socio-economic inequalities, family planning, nutrition, smoking and alcohol. Like Lewis, he maintains that the major developments in public health around disease prevention and epidemiology took place elsewhere, with MOsH failing to provide the necessary leadership.^21^

This negative view of the work of the MOsH has been challenged. Martin Gorsky argues that, although there is some basis to the thesis that public health services lost their focus in the twentieth century, largely due to the increasing emphasis on curative rather than preventive services, MOsH were able to adapt and redefine their role.^22^ In his study of public health services in Bristol and the South West during the interwar period, Gorsky pointed out that the MOsH were especially active in matters concerning malnutrition. Moreover, whilst the MOsH did follow the national lead on health education to some degree, most of their efforts (including film showings and travelling exhibitions) came from local initiative.^23^ A similar picture was found by John Welshman, who looked at the activities of the MOH in Leicester from 1900 to 1974. He established that the MOH was an active force, especially so with respect to health education. A health education department in the city was established in the 1950s, and, although some of the material was paternalistic in tone, it produced campaigns on significant issues such as diphtheria immunisation and dental health.^24^ Another local study, this time of Aberdeen by Lesley Diack and David Smith, also points to important and even innovative work in health education by the MOH. In the 1950s and 1960s, Aberdeen’s MOH, Ian MacQueen, was especially active in health education, with over 1000 public talks a year being given by his department. MacQueen was also quick to recognise the value of the mass media for communicating health messages on disease prevention.^25^ Taken together, this body of work would suggest that the MOsH were far from moribund by the post-war period.

It was not just MOsH, however, that were engaged in health education. The Ministry of Health, other government departments, voluntary organisations and the Central Council for Health Education (CCHE) all carried out health education campaigns from the 1900s onwards. There was, for instance, special concern about the health of girls and young women, at home, at school and in the workplace.^26^ Hilary Marland and Vicky Long note that health education for women at this time revolved around hygiene, diet, exercise and recreation as well as anxieties about the impact of work on female health.^27^ Over the course of the early twentieth century, Lewis suggests that public health moved away from environmental concerns and towards ‘personal prevention’ aimed at averting disease and promoting health in individuals through clinics. There was a strong emphasis on personal hygiene, often directed at working-class mothers with the aim of reducing infant mortality and improving the health of the nation.^28^ As Welshman notes, many of these early efforts in health education were imbued with the promotion of morality and good citizenship.^29^ There were also attempts to provide health education and co-ordinate efforts at the national level. The CCHE was established in 1927 to provide leadership in health education. The Council was funded by contributions from local authorities and was in perennial financial difficulties, which hampered its effectiveness.^30^ Some nation-wide campaigns were produced, such as those on venereal disease during the Second World War, and vaccination for diphtheria and polio in the post-war period.^31^ The CCHE also implemented several of the early anti-smoking campaigns, although this was towards the end of the organisation’s existence.^32^ Health education remained primarily a local responsibility until the late 1960s. In 1964 the CCHE was judged to be ineffective by the Cohen Committee on Health Education, and it was replaced by the Health Education Council in 1968.^33^ However, the Cohen Committee was not just critical of the CCHE: the committee argued for a new approach to health education, one that involved much greater use of the mass media.^34^

The use of the mass media in the latter third of the twentieth century was a new departure for health education, but it also drew on techniques developed in earlier decades. Visual and audio-visual methods of communication, including posters and films were utilised by health educators from their introduction in the 1900s.^35^ Technological developments made the reproduction of images easier and cheaper, opening a wide range of channels to advertisers and others seeking to communicate their message to an increasingly visually literate audience. Bringing together objects and images to form exhibitions was a powerful way of communicating with the public about health-related topics. Medical museums, particularly those featuring anatomical models, were common throughout Europe and North America during the late nineteenth century and early twentieth century.^36^ More temporary presentations of health exhibits could also be found in popular expositions, and in addition there were dedicated public health exhibitions, such as the International Health Exhibition held in London in 1884, the Popular Hygiene Exhibition convened in Mexico City in 1910, and the International Hygiene Exhibition in Dresden in 1911.^37^ As Julie K. Brown points out, by the assembling of exhibits and public audiences, health exhibitions were part of a new visual culture that began to flourish in the early twentieth century.^38^

By the middle of the century, exhibitions fulfilled a variety of roles: they were trade fairs, sites of aesthetic discourse, projections of national identity and vehicles for creating a more enlightened public. Indeed, exhibitions were one of the key ways in which the post-war British state sought to talk to its citizens. In the late 1940s and early 1950s, several government-sponsored exhibitions took place in Britain. Perhaps the most famous of these was the Festival of Britain in 1951, which contained a health dimension.^39^ Sir Fife Clark, Director General of the Central Office of Information between 1954 and 1971 (the UK government’s dedicated department for communicating with the public) stated that ‘The appeal of the actual, especially working exhibit, is a powerful one.’^40^ It is, therefore, no surprise that MOsH organised health exhibitions as a way of communicating with the public. An analysis of the content of these exhibitions can tell us much about the changing nature of public health from the late 1940s to the early 1970s.

## 2 The Content of MOsH Exhibitions

**Figure 1:**
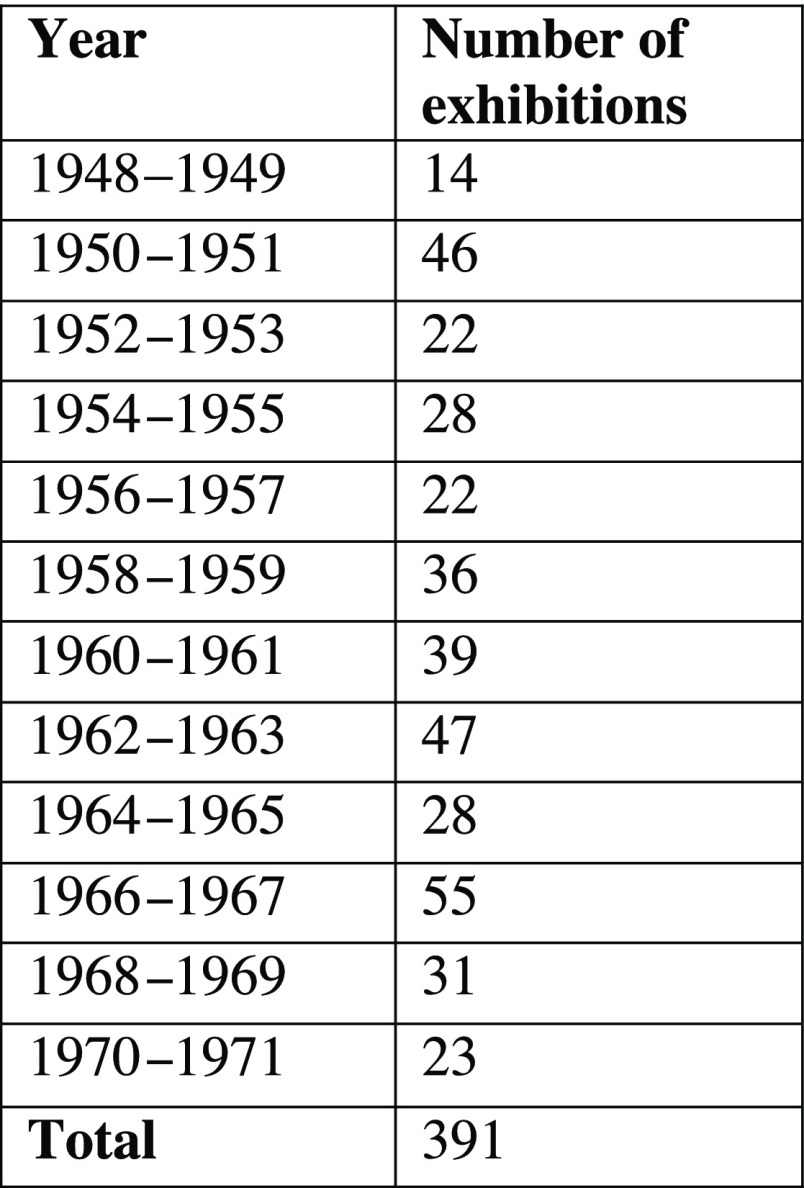
Years and numbers of exhibitions.

**Figure 2:**
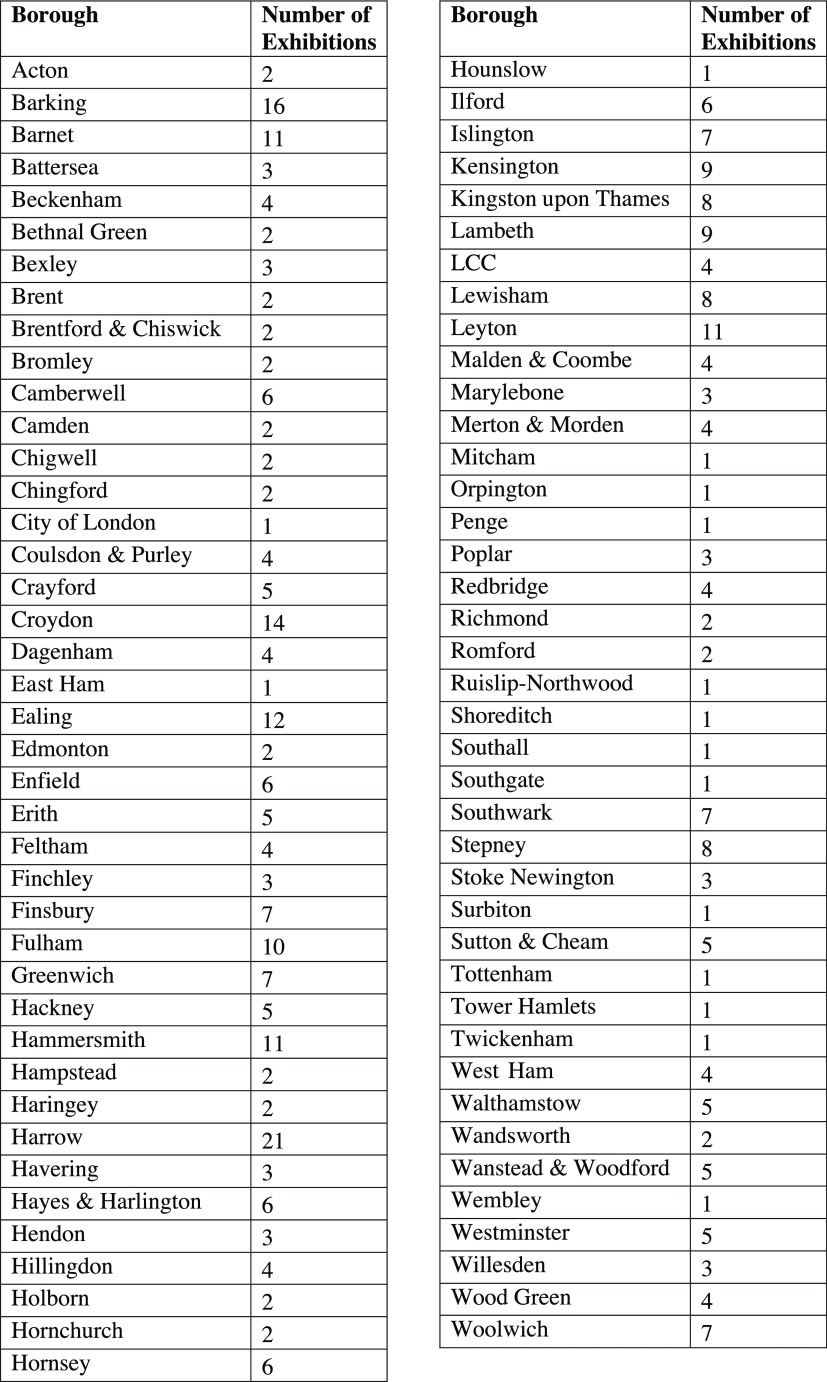
Boroughs and total number of exhibitions held, 1948–71.

**Figure 3:**
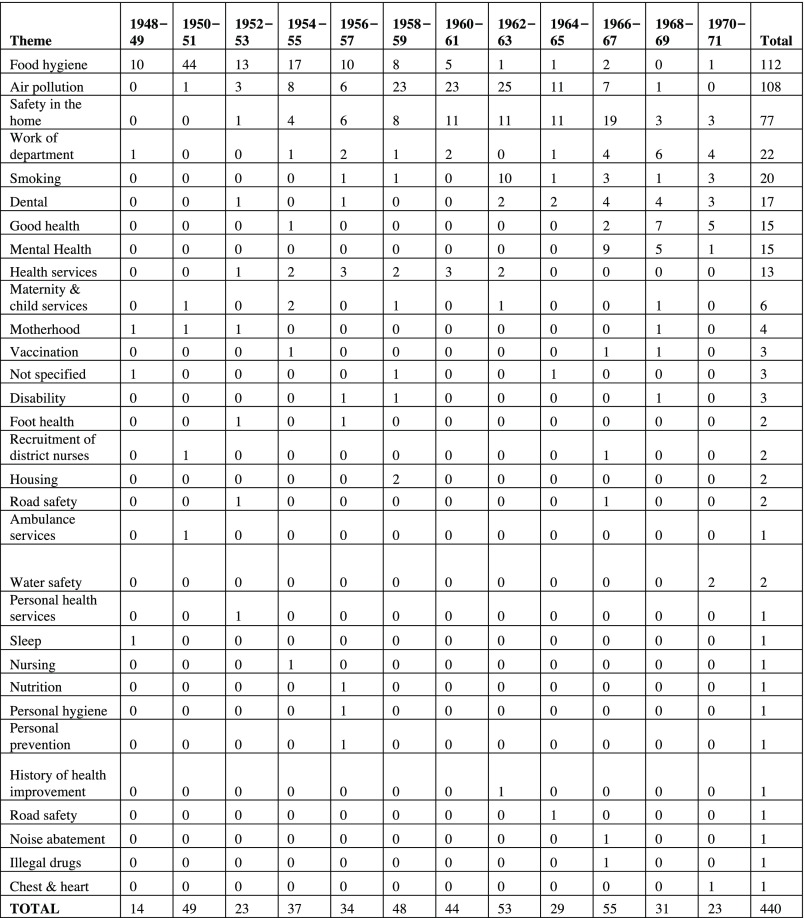
Themes of exhibitions, 1948–71.

**Figure 4:**
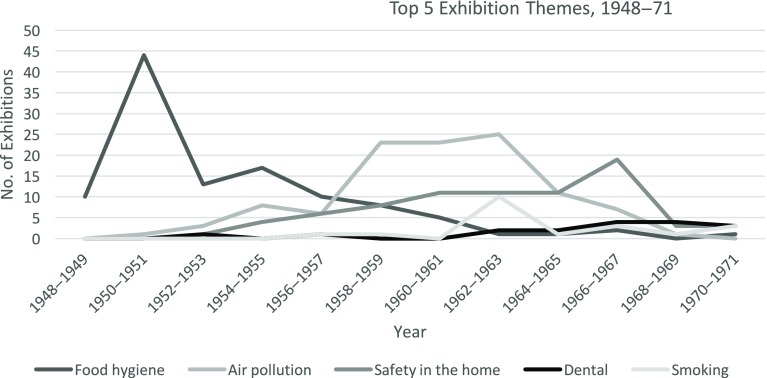
Top 5 exhibition themes, 1948–71.

During the period from 1948 to 1971, the London MOsH organised 391 exhibitions (Figure 1). Almost all MOsH appear to have held an exhibition during this time.^41^ A handful of MOsH, like the officers for Leyton, Fulham and Ealing, put on an exhibition almost every year; in other boroughs, like Shoreditch and Twickenham, exhibitions were rare (Figure 2). There does not appear to be any direct relationship between the affluence of the borough and the frequency with which exhibitions were held. The boroughs that held the most exhibitions were quite diverse. Fulham had a similar class make-up to the London average; Ealing was more affluent, with a larger concentration of people in classes 1 (professionals) and 2 (intermediate occupations), whereas few professionals lived in Leyton. Some boroughs with relatively large concentrations of people in classes 1 and 2, like Westminster and Hampstead, rarely held an exhibition, but having a significant working-class population did not necessarily mean more exhibitions: Bethnal Green held only two exhibitions despite almost a quarter of its population being categorised as in unskilled occupations.^42^ The role of class in MOsH exhibitions is discussed in greater detail below, but the number of exhibitions held in a borough probably tells us more about the preferences of the MOsH than the socio-economic make-up of the area. What is more revealing, however, is the topics covered by exhibitions and how these changed over time. The most common theme, certainly in the early part of the period, was food hygiene. Of 440 exhibitions held between 1948 and 1971, (some double-counted, as they contained more than one theme) just over a quarter (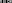) were on food hygiene (Figure 3). From the mid-1950s, however, food hygiene started to drop away as a concern, to be replaced by air pollution, safety in the home, and smoking and lung cancer (figure 4). A more in-depth look at the content of MOsH exhibitions can help explain how and why such changes occurred.

Ensuring the preservation of standards in food hygiene had long been one of the responsibilities of the MOsH, but there were other reasons why the issue was so prominent at this time. Although there were relatively few deaths from food poisoning, it did seem to be on the rise. The Chief Medical Officer noted in 1948 that the number of reported outbreaks of bacterial food poisoning had risen from 422 in 1945, to 962 in 1948.^43^ The gradual end of rationing, the introduction of new foodstuffs and changes in animal husbandry, all contributed to a rise in incidences of food poisoning. Over the period 1946–65 there were nearly 100 000 cases of food poisoning.^44^ MOsH were concerned with establishing good food hygiene standards in commercial premises, but also in the home. This was important at a time when few domestic kitchens contained a refrigerator: in 1956, just 8% of British homes had a refrigerator or freezer, rising to 38% by 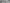.^45^

**Figure 5:**
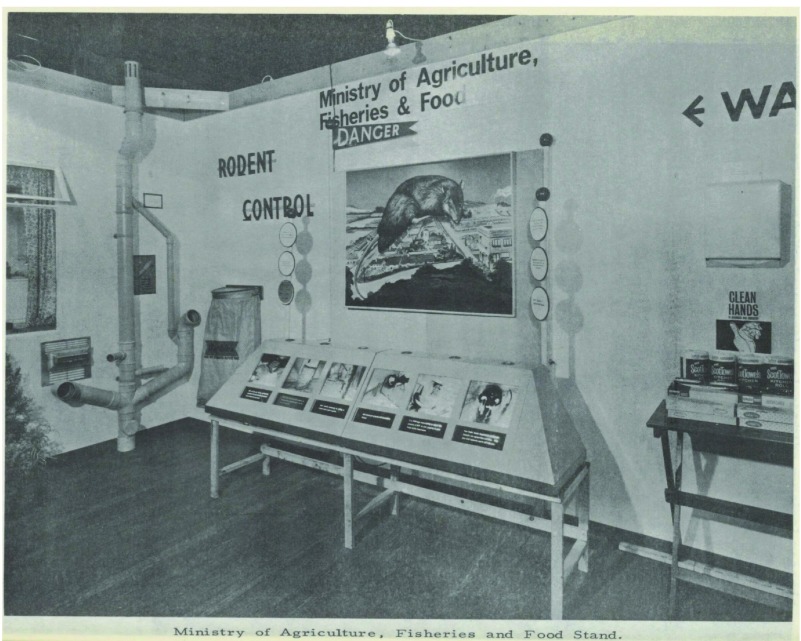
Photograph of exhibition organised by MOH for Hampstead, 1962, with a prominent image of a rat. Source: Report of the Medical Office of Health for Hampstead, 1962. No page number. Permission: Licenced under Creative Commons Attribution 4.0 International Licence.

**Figure 6:**
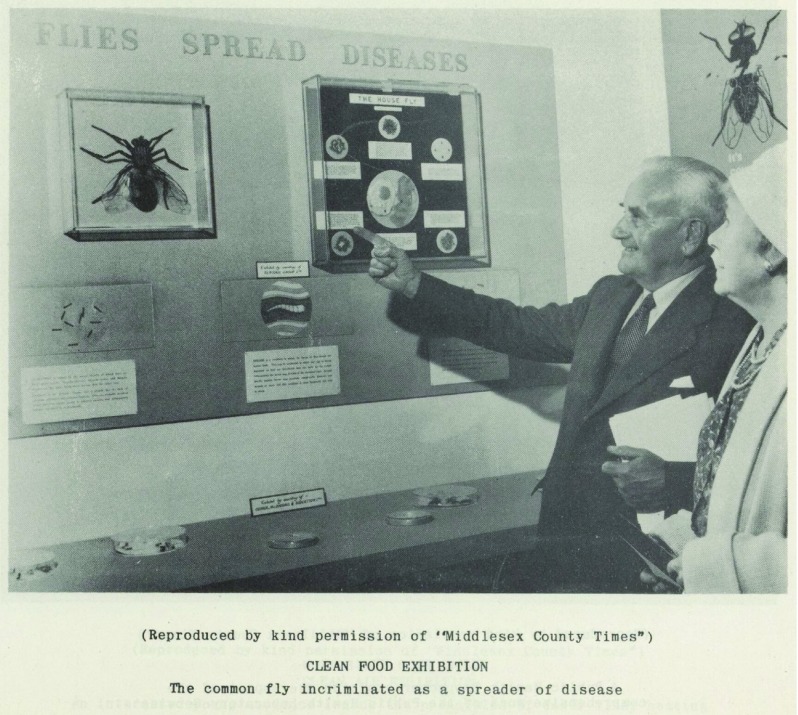
Photograph of exhibition organised by MOH for Ealing, 1961: The common fly as a spreader of disease. Source: Report of the Medical Office of Health for Ealing, 1961. No page number. Permission: Licenced under Creative Commons Attribution 4.0 International Licence.

Like the exhibition in Ealing described so vividly by the reporter on Woman’s Hour, many MOsH also made use of exhibits demonstrating bad practice to communicate a message about good food hygiene. A much-favoured tactic appears to have been the display of pests, including rodents and insects (Figures 5, 6). The MOH for Erith’s food hygiene exhibition held in April, 1949 featured a ‘display of live rodents’ which ‘successfully demonstrated the importance of food protection’.^46^ In 1955, in Wanstead and Woodford, the MOH reported that the stand at the exhibition ‘with live rats and mice was a great attraction to the younger folk’.^47^ Although such exhibits clearly appealed to ‘younger folk’, there were two main targets for food hygiene messages: those working in food production or sales, and the housewife. The gendering of such efforts was often explicit. One innovative MOH in West Ham presented each female visitor to the exhibition with a nail brush bearing the slogan ‘Clean Hands, Clean Food’.^48^

**Figure 7:**
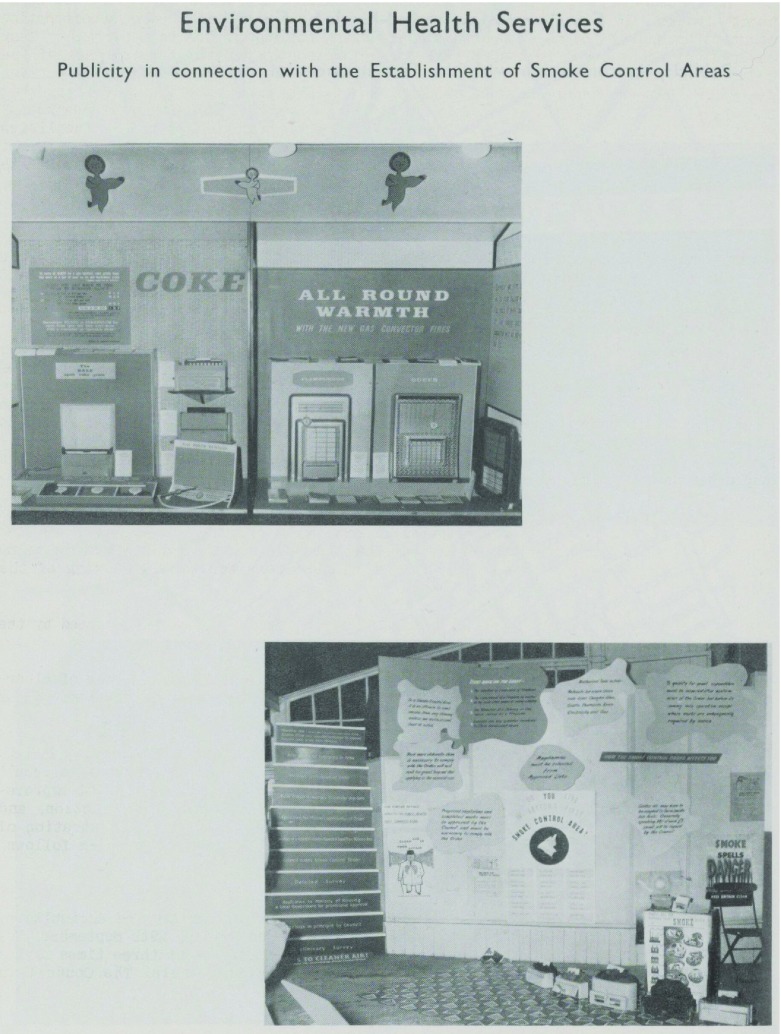
Photographs of exhibition on air pollution and smokeless fuels organised by MOH for Leyton, 1961. Source: Report of the Medical Officer of Health for Leyton, 1961, 20. Permission: Licenced under Creative Commons Attribution 4.0 International Licence

Yet, food hygiene was not the only existing danger to public health that assumed new importance in the post-war era. The environment had long been thought to pose a threat to health. The introduction of sewers, clean water and drainage during the nineteenth century resulted in the removal of one kind of environmental cause of ill-health, but air pollution was still a concern. Following the Great Smog in 1952, the Clean Air Act was introduced in 1956. The Act required those living in smoke control areas (which included all of London) to burn smokeless fuels in their homes.^49^ MOsH exhibitions after 1956 often dealt with the issue of clean air, sometimes collaborating with utility providers and commercial enterprises to demonstrate the use of smokeless fuels and appliances (Figure 7). Indeed, the MOsH appeared to have been increasingly concerned with what went on in homes, and not just in relation to food hygiene in kitchens, or the burning of smokeless fuels in living rooms. Safety in the home, and the prevention of domestic accidents was an increasingly common theme of exhibitions during the mid-1950s and early 1960s. Some MOsH went to considerable effort to get the message across. In 1951 the MOH for Woolwich exhibited a ‘Trouble House’ in the department’s waiting room. The model consisted of ‘an open-sided house, the rooms and contents being in full view. Each room was fully furnished and complete with model figures staged in a manner to demonstrate the hazards created by the misuse or faulty arrangement of household equipment.’^50^ Other MOsH sought to reach as many people as possible. In 1957, and again in 1959, the MOH for London County Council held an exhibition on home safety in Charing Cross station that attracted over 27 000 and 23 000 visitors respectively.^51^ Yet, some MOsH were also sensitive to the potential intrusion into the home that such messages represented. In 1957 the MoH for Leyton played upon the popular notion that an ‘Englishman’s home is his castle’ displaying an exhibit that ‘took the form of a castle’ but ‘has he [the homeowner] a fifth column within, the fifth column being does he take all precautions against accidents in the home’.^52^

Reaching in to the homes of the public was not a novel task for MOsH, but what was new was the fact that such efforts were targeted not only at the working classes, but at the more affluent too. Previous intrusions into the home, especially in the early twentieth century, had focused on the health of working-class mothers and their babies.^53^ Motherhood was still a concern for some MOsH in the post-war period. For instance, in 1949, the MOsH for London County Council held two exhibitions: one on ‘mothercraft’ and the other on maternity and child welfare services.^54^ Yet, there were signs of a different approach to that offered in the pre-war decades. After 1948 there appears to have been more emphasis on the services available, and not just to mothers and babies, but to all citizens. This was, of course, important in the age of the new NHS, when health educators wanted to communicate to the public the types of services on offer and to give information on how to access these. But there were broader aims at work too: communication about health services also helped to foster a sense of health citizenship.

Notions of good citizenship could be found in pre-war health education, but after the establishment of the NHS, this took on a different dimension.^55^ The provision of health services was part of a wider package of social rights guaranteed by the expanded welfare state.^56^ MOsH wanted to communicate these to the public. The MOH for East Ham organised an exhibition on ‘the part played by the health services in the life of the community’ during civic week in October, 1949. There were displays on ‘the continual need for good standards of personal hygiene’ but also ‘colourful and attractive exhibits of the work of the mental health occupation therapy centre, a symbolic representation of the activities of the home help and home nursing services and arrangements of prepared foods illustrating ideal meals for growing children at various ages’.^57^ Such displays were not just confined to the early years of the NHS. Throughout the 1950s and 1960s, and even into the early 1970s, MOsH organised exhibitions under the title of ‘Welcome to citizenship’.^58^ Often aimed at young people, these exhibitions told visitors about the services provided by their local health department. Health citizenship was not, however, just about rights, but also involved responsibilities. These included making prudent and effective use of health services, but, as health became linked to behaviour, also embraced a duty to remain well.^59^

Such a shift towards lifestyle-related health can be observed in the changing content of MOsH’s exhibitions. By the mid-1950s, new concerns seemed to come to the fore. Chief amongst these was tobacco smoking. The discovery, in 1950, by Richard Doll and Austin Bradford Hill of a causal link between smoking and lung cancer took time to have an impact on public health policy and practice. It was not until the publication of the Royal College of Physicians Report on *Smoking and Health* in 1962 that the link between smoking and lung cancer was universally acknowledged.^60^ Webster was critical of the MOsH for being slow to take up efforts to combat cigarette smoking, but Virginia Berridge has shown that Medical Officers were responsible for the first anti-smoking campaigns in the early 1960s.^61^ Indeed, some enterprising MOsH were becoming concerned with smoking before this period. In 1957 the MOH for Croydon mounted an exhibition on the dangers of smoking, targeted particularly at young people. Moreover, he recognised that this new threat to public health might require fresh approaches, commenting that: ‘It is difficult for those who have been in the field for a long time to change from comfortable routines and didactic methods; the change spells uncertainty and insecurity; but change also means progress.’ He was optimistic that ‘Research has shown that in spite of wide differences in kinds and rates of learning, there is a capacity for significant change in ideas, attitudes and modes of behaviour at all ages.’^62^ Indeed, a survey of the public’s views on ‘health propaganda’ conducted by the MOH for Camberwell in 1959, found that people wanted to know more about smoking and the dangers it posed to health, and they also wanted more money to be spent on health education on the topic.^63^

The kind of behaviour change required by the establishment of a link between smoking and lung cancer, and thus the kinds of health education messages and media, were different to what had gone before. Although past health education efforts by MOsH had been directed at changing behaviours (on food hygiene say, or spitting in public), the types of behaviours to be altered, and the public health authorities’ understanding of these, was shifting. Health education was no longer about stopping people from actions that spread disease, but rather about getting them to change their behaviour to reduce the risk of developing a chronic condition. Underpinned by epidemiological evidence that demonstrated the harmful nature of specific actions on a population level, health education could now target certain behaviours and the individuals that indulged in them. Smoking, for example, was one of the ‘ways of living’ described by the renowned epidemiologist Jerry Morris. These were comprised of a broader set of individual behaviours that included things like diet, physical activity and alcohol consumption that appeared to pose a danger to health.^64^ The appearance of what later became known as ‘risk factors’ for the development of chronic disease implied a new kind of health education to be delivered in a new way.^65^ If behaviour was both cause and remedy for the most pressing public health problems, then health education needed not just to inform, but also to persuade. To do so effectively, the MOsH had to get closer to the people that they wanted to change.

## 3 The Techniques of Display

An examination of the ways in which MOsH attempted to present their health education messages by means of exhibitions suggests that they were beginning to take account of such shifts. A significant amount of thought, time and effort went in to planning exhibitions, designing the exhibits and staffing the shows. As the MOH for Croydon remarked, exhibitions ‘involved an enormous amount of work, but were used as fully as possible to “teach without tears” ’.^66^ Exhibitions, P.K. Hendy, an exhibitions officer at the CCHE advised, must aim: ‘(a) to attract attention, (b) to interest and stimulate and (c) to create a desire for action’.^67^ To achieve these goals, he suggested, exhibitors should draw on as wide a range of media and techniques as possible. MOsH certainly took this message to heart. Exhibitions included photographs, films, posters, leaflets, objects and equipment, models and recreations, dioramas, paper transparencies, battery operated displays, quizzes and competitions, question and answer sessions, and demonstrations. Sometimes MOsH borrowed exhibits from the CCHE (who offered to loan material to local public health departments from 1949–56), or from other boroughs.^68^ MOsH also showed films produced by other agencies, such as the Central Office for Information and the Gas Council, but often they created their own resources.^69^ Exhibitions differed in their scale and duration. Some were very large, comprising of multiple exhibits and lasting many days, such as that organised by the MOH for Leyton in 1957.^70^ Others were smaller in scale and scope, but, as the MOH for Camberwell asserted, small exhibitions were not without their advantages: ‘Small portable single-topic exhibits which can be taken from place to place and shown for short periods in various parts of the area’ were ‘probably more effective’.^71^

Considerable thought was given to the location of exhibitions, often with the aim of reaching a large audience, or one felt to be most in need of health education. The MOH for Stepney, for instance, decided to hold an exhibition in 1950 outdoors, as ‘it would probably be more successful to take the exhibition to the public rather than expect the public to attend an indoor exhibition in a public hall. It was also felt that in this way the educational effect of the campaign would better reach those sections of the public who were probably most in need of enlightenment’.^72^ The department hired a mobile cinema van and toured round the borough to take their message to the people. Other outdoor locations made use of by MOsH included parks and shopping centres.^73^ The MOH for Wembley felt that by utilising shop fronts the exhibition ‘undoubtedly was seen by thousands more people than would have visited an exhibition held in a hall’.^74^ Another tactic was to hold an exhibition at the same time as a popular local event. The MOH for Enfield organised an exhibition to coincide with the Enfield Show in 1956, thereby ‘captur[ing] the interest of the fairly large and representative section of the public’.^75^ Many MOsH used the waiting rooms in health clinics, employment bureau and the public health department itself to mount displays.^76^ Other local government facilities, such as Town Halls and libraries, were also exploited.^77^ The intention behind the use of this diverse range of locations appears to have been to reach the public where they were, rather than encourage the public to come to a stand-alone exhibition. Coupled with the diverse methods and the media used in exhibitions, it would seem the MOsH were making a genuine effort to connect with the public in a variety of ways and settings.

## 4 The Relationship between the MOsH and the public

Reaching out to the public rather than expecting them to be willing recipients of health messages was bound up with a broader change in tactics and the underpinning philosophy of health education in this period. From the early days of the NHS, some MOsH were aware that they were to play a greater role in health education. In 1949 the MOH for Edmonton stated that ‘Health Education should now become a prominent feature of the Council’s Services to the people. Up to the present it formed an accidental corollary to other functions of your officers …Now, education of the public in the circumstances of health is a duty which the Council must no longer neglect.’^78^ That same year, the MOH for Southgate contended that ‘Money spent on health education is never wasted; results may not be immediately obvious, but unless the work is misdirected, benefit will most definitely accrue.’^79^ Other MOsH, whilst prepared to take on more health education work, felt that this could be an ‘up-hill task’ and that ‘The general public do not, perhaps, sufficiently recognise that effective official action is only possible with their co-operation.’^80^

Indeed, by the later 1950s, there was a realisation amongst some MOsH that health education needed to do more than simply inform the public. The MOH for Croydon, Dr S.L. Wright, was especially interested in taking a new approach to health education. In 1957 he asserted that ‘the aim of health education is to help people to achieve health by their own actions and efforts and develop a sense of responsibility for it as individuals and as members of families and communities’.^81^ A year later, he noted that ‘we realised that to inform alone would not succeed and that we must make even greater efforts to interest and persuade’. Wright concluded that ‘Our main purpose in health education is not to provide services for the individual, but rather to help him remove harmful habits from his behaviour and adopt by himself a healthy attitude towards life – to depend more on his own activities for his own well-being.’^82^ Education still had a role to play, but, as the 1950s gave way to the 1960s, the purpose of information-giving began to change. In 1965 Dr J. Kerr Brown, the MOH for Greenwich, commented that ‘there are many aspects of modern health thinking which depend upon individual decisions …Problems of smoking, dental caries, obesity, lack of exercise, casual promiscuity or the more dramatic forms of self injury such as drug taking are current examples.’ Brown continued ‘In these and other matters, including home accident prevention, the aim of health education is to achieve a climate of opinion where indulgence in anti-health activities is viewed with the same distaste as infrequent bathing, spitting, etc., are regarded to-day.’^83^ Although the parallels Brown drew with earlier health education campaigns such as stopping people from spitting in public might suggest some continuity with interwar and wartime efforts, what was different about the approach taken from the late 1950s and early 1960s was its wider view of behaviour and the role of information in shaping this. For instance, the MOH for Redbridge suggested that, although ‘The notion of health education being a series of prohibitions still prevails in some quarters’, it was ‘our task to give a lead in creative thinking. Not so much in imparting information but more to teach the techniques and methods of knowing where to look for it, and to help people solve their own problems.’^84^ Such an approach indicated a changed role for both health educators and the public: health education was increasingly concerned with persuading individuals to change their behaviour and lead more healthy lives, rather than just providing information about how to guard against certain diseases, or how to access services.

The extent to which the public were willing and able to take on these messages was a matter of concern for MOsH. Some saw the public as apathetic. John Landon, the MOH for Bexley, Erith and Crayford, for instance, remarked in 1952 that there was ‘considerable public apathy in regard to film shows, lectures and exhibitions’.^85^ Contemporary public health practitioners and policy makers were worried about public apathy in other areas too, such as vaccination uptake. As Gareth Millward suggests, ‘apathy’ was to some extent an artificial construction, a convenient way for the authorities to lump together a set of wider public reservations about vaccination under one heading.^86^ Likewise, the public may have appeared ‘apathetic’ about health education for a whole host of reasons: because the presentation was uninspiring, because the message being communicated was unappealing, or already familiar. Indeed, there was a fear amongst MOH that health education ‘preached to the converted’: reaching those who were already most amenable to its messages.^87^ At the same time, there was also some optimism that the exhibition was a mechanism that could bring health education to a wider audience. Rowntree stated in *Health Education* that ‘A criticism often levelled at the health educator is that he “preaches to the converted”, but through the medium of the exhibition contacts are often made with the “unconverted” who attend as part of the anonymous “general public”.’^88^

Whilst some MOsH expressed a desire to reach as wide an audience as possible by means of their exhibitions, others targeted their efforts at certain groups. The MOH for Stepney, for instance, wanted to communicate with ‘those sections of the public who were probably most in need of enlightenment’.^89^ Although he did not spell out exactly who these members of the public were, other MOsH were less coy. In 1959 the MOH for Camberwell conducted a survey on the public’s views of health education. He was disappointed with the completion rates amongst semi-skilled and unskilled workers, ‘who comprise about 30% of the population’ but, ‘only 11% who completed questionnaires were in these groups, yet there is no doubt that it is here that the need for health education is greatest’.^90^ Other groups that came in for special attention included children and women. As noted above, housewives were one of the key targets of food hygiene exhibitions and mothers were the central audience for exhibitions on parenting. Moreover, women and children represented the traditional targets of public health services, but there were signs that a new group was also coming in for increased attention. In 1961 the MOH for Camberwell stated that ‘Health education is of particular importance among the immigrant population, many of whom are unused to the standards of living which prevail in this country.’^91^ During the late 1950s and early 1960s, immigrants from the West Indies and the Indian sub-continent began to arrive in the UK. As Roberta Bivins points out, these newly arrived groups came in for additional scrutiny by public health officials because of the supposed risk that they posed in transmitting diseases such as TB and smallpox. The threat to public health, however, was small and was hard to disentangle from broader public and political fears about mass immigration.^92^

Despite their desire to target groups thought to be in the greatest need of health education, such as the working classes, women, children and immigrants, MOsH also wanted to appeal to a general audience too. The MOH for Leyton commented that he aimed to reach the public ‘en-masse’. This was a move reflected elsewhere within public health services, such as in the development of survey methods that aimed to capture a representative cross-section of the population from all socio-economic groups.^93^ As an epidemiological view of the health of the population as a totality began to develop, public health services were increasingly concerned with the health of everyone, not just women or the working classes.^94^ For key figures within public health policy and research like Morris, the differences in the occurrence of conditions such as lung cancer lay in individuals (and their behaviour) and cut across social classes, occupations and regions.^95^ As a result, public health practitioners needed to contact a much larger constituency than they had done in the past.

There is some evidence to suggest that by means of the exhibition MOsH were successful in reaching a wider public. Attendance at exhibitions varied widely, but large audiences were not unusual. More than 23 000 people visited an exhibition that was organised as part of the Enfield Show in 1966; 15 000 people attended an exhibition in Hayes in 1953; 9000 people saw the MOH exhibition in Acton in 1950; 6000 people went to the one in Barking in 1951; and 5000 to Islington in 1954. Yet, some exhibitions appeared to have fared less well. The MOH for Battersea reported an attendance at his exhibition in 1950 of 500 adults and 200 children, figures which he thought were ‘rather disappointing’.^96^

Even when attendance at an exhibition was thought to be ‘discouraging’ some MOsH could find comfort in the ‘interest and enthusiasm displayed by those who attend’ which served ‘as a spur to further effort’.^97^ Judging the public’s reaction to MOsH exhibitions from MOsH reports is of course problematic, but even viewed through this partial and slanted lens glimpses of what the public thought of the exhibitions, and public health services more broadly, can be discerned. The exhibition in Acton that had attracted an audience of over 9000 people was judged by its creators as ‘an undoubted success’ in which ‘the public showed a very deep interest’.^98^ Certain exhibits appeared to have been especially popular. Live rodents (particularly rats) and insects were favoured by the viewing public. The MOH for Hornsey noted in 1953 that ‘Schoolchildren found the display of live insect parasites the most interesting exhibit.’^99^ ‘Live’ exhibits appealed to older visitors too. The MOH for Woolwich mounted a ‘display of diseased meat specimens’ which ‘proved of unusual interest’.^100^

It is tempting to see in the popularity of such exhibits a prurient element: provoking disgust could be entertaining as well as a tactic for communicating health messages.^101^ However, other interactive exhibits without a shocking component also proved popular. Some of these concerned the display of new technologies and equipment. At the MOH for Hornsey’s 1953 exhibition ‘Special interest was shown in the safety devices now enforced in respect of electric, gas and oil heating apparatus.’ Yet, for the MOH, this exhibition was also an occasion for the display of public ignorance. He reported that ‘People who had installed special grates for all-night burning did not seem to realise that they were contributing to the “smog” when they banked the fires up at night with dust and waste material; only a few of them habitually burned smokeless fuels in these grates.’ Moreover, ‘The “smog” mask and its limitations were explained to the public and some of the elderly people showed impatience in the inability to do away with “smog” altogether but still expressed their wishes to have open coal fires so that “they could see the flames”!’^102^

**Figure 8:**
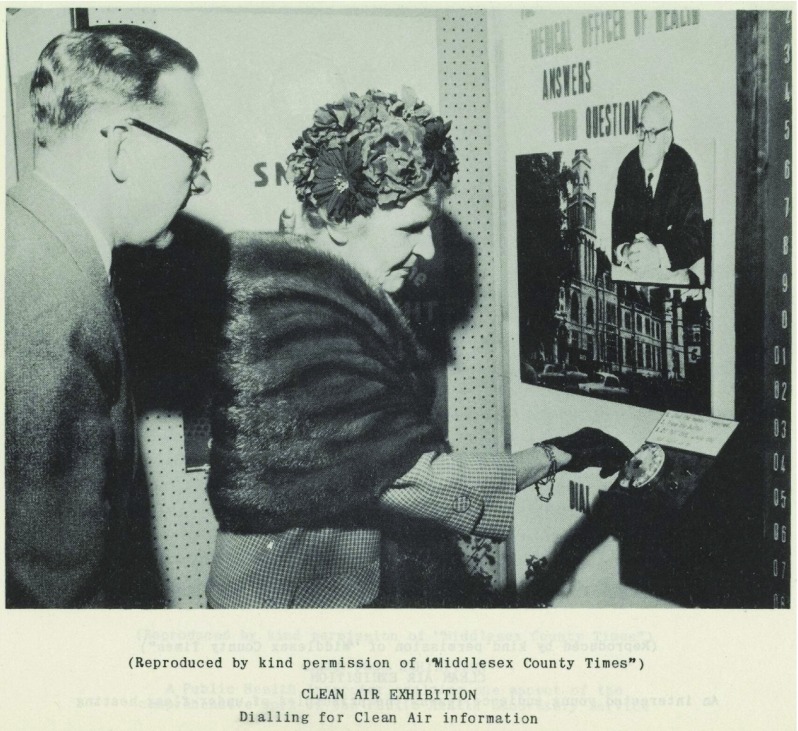
Photograph of automatic question and answer device used in exhibition organised by MOH for Ealing, 1961. Report of the Medical Officer of Health for Ealing. Permission: Licenced under Creative Commons Attribution 4.0 International Licence.

Exhibitions brought citizens and the MOH into direct contact, exposing differences between the activities and beliefs of the public, and those held by public health practitioners. A survey administered by the MOH for Camberwell in 1959 to ascertain the public’s view of ‘health propaganda’ provided a free-text box for members of the public to enter their ‘remarks’. The MOH noted that these covered a ‘very wide range and were frequently not relevant to health education’. Some health concerns were raised, such as the need for more research on cancer, but the comments ‘often reflected the particular grievances of the respondents, such as complaints about litter on waste land, and inadequate housing’.^103^ Similarly, at an exhibition organised by the MOH for Stepney, in 1950, questions asked by members of the public were frequently ‘irrelevant and turned on housing and personal economic difficulties’.^104^ The raising of such concerns, and their dismissal by MOsH as being of the ‘wrong’ sort suggests not only that the public had a hard time disentangling the division of responsibilities between MOsH and the borough council, and between national and local government, but also between the wider socio-economic causes of ill-health and the behavioural elements MOH exhibitions were concentrating on. Whilst some MOsH were acutely aware of the impact of social conditions on health, this did not appear to figure in their interactions with the public. Question and answer sessions, and the presence of public health officials at exhibitions, were not always as open as the format might have suggested. In Stepney the exhibition staff were provided with a list of 30 pre-selected questions and answers to be used in ‘slack moments, when public questions were not forthcoming’. At this point, ‘staff members in the crowd put up questions from the list, which were answered by the Speaker, and were usually followed up by supplementary questions from the public’.^105^ The MOH for Ealing went one step further by displaying an automatic question and answer device ‘which proved particularly effective in providing the public with information’ (Figure 8).^106^ In Hampstead the MOH created a ‘juke box’ ‘which in addition to giving information by question and answer on specially made records, also allowed the selection of some of the more popular musical records and this was much appreciated by some of the visitors’.^107^ These methods, whilst innovative, did not allow for much direct interaction or genuine questioning of public health officials by the public. Nonetheless, the fact that MOsH were beginning to take an interest in what the public thought was an important shift. In 1952, the MOH for Barking, for instance, held an ‘open forum’ where ‘members of the public were able to ask a panel of speakers questions concerning “local government and the citizen”’.^108^ A willingness to engage with the public was crucial in the shift towards focusing on lifestyle-related chronic disease, as this required a much greater role for the public in the preservation and maintenance of their health and that of others.

Increased interest in the public on the part of the MOsH can also be seen in a growing concern with the effectiveness or otherwise of exhibitions and other methods of health education. At first, many MOH seem to have assumed that their efforts were effective, or at least they did not scrutinise them too carefully. For example, the MOH for Wood Green, writing in 1955, was ‘more than ever satisfied that, in health education, we have a weapon which is both economical and effective’ but he offered no evidence to indicate that this was the case.^109^ Such an assumption was common among both MOsH and national bodies and officials tasked with health education. As Berridge points out, the systematic evaluation of health education efforts was rare until the 1980s.^110^ Yet, some MOsH were concerned about the impact of their health education work. In 1951 the MOH for Wembley noted that it was hard to tell whether his food hygiene exhibition had had any effect: ‘as with many facets of public health work there is unfortunately no “yard stick” by which appraisal of efforts of this kind can be accurately gauged; one can only speculate’.^111^ Similarly, the MOH for Ealing stated that ‘It is very difficult to evaluate the full effects of such an Exhibition, but I feel confident that many of the visitors now have a much clearer understanding of the Council’s aims and intentions.’^112^ Other public health officials, like Dr H.D. Chalke, MOH for Camberwell, appeared to have been more willing to tackle the question of the effectiveness of health education head-on. His survey on health education in the borough, carried out in 1959, and later published in the journal for MOsH, *Medical Officer*, was an extensive attempt to build up a picture of the public’s view of different methods of health education and their impact. What Chalke found was a cause of some dismay. When asked to rank different methods of health education in order of preference, survey respondents placed exhibitions ninth out of eleven choices. This, commented W.M. Clunie Harvey, the MOH for Friern Barnet, Southgate and Wood Green, was hardly surprising, since ‘exhibitions, unless they deal with an urgent, topical problem, have singularly little effect upon the public’.^113^ In contrast, television programmes were regarded by survey respondents as the most effective form of health education, followed by films, talks in schools, and radio programmes. This led Chalke to conclude that ‘it would seem that since the public are responsive to instruction by television, health magazines, and modern commercial advertising, a great deal more use should be made of them’. In particular, ‘The popularity of television as a health educational medium cannot be overlooked; it must surely serve as a guide to future activities in health promotion.’^114^

Chalke, of course, was right. By the late 1950s, and certainly by the early 1960s, the mass media could no longer be ignored as an important vehicle for health education. In 1964 the Cohen report on health education recommended a greater role for mass media in promoting good health, noting that there was a ‘special’ role for television in this area.^115^ Television ownership increased dramatically in this period: in 1946 very few homes in Britain had a TV; by 1956, about one third of homes did; by 1966 almost all homes had a set.^116^ By the 1970s and 1980s, health educators were making use of a wider range of techniques, often borrowing from commercial advertisers, to persuade individuals to change their behaviour to improve their health and that of the public more broadly.^117^ As the Cohen report stated, ‘Health education must do more than provide information. It must also seek to influence people to act on the advice and information given, and must seek to counteract the pressures which are inimical to health.’^118^

## 5 Conclusion

The Cohen report marked official recognition of a changed role for health education in persuading rather than simply informing the public, but, as the work of the MOsH on exhibitions in the 1950s and 1960s demonstrates, there was already movement in this direction. Some MOsH were aware that, as the major threats to public health shifted, so their response to these also needed to be altered. The immediate post-war period was dominated by concern about food hygiene and communicating information about the provision of services, especially those available to mothers and children. In many ways, these were the traditional concerns of public health services, central to the work of the MOsH since the early twentieth century and earlier. But, by the 1960s, MOsH and their exhibitions featured new concerns, such as smoking and safety in the home. With these novel issues came a fresh emphasis on attempting to influence individual behaviour, rather than simply aiming to inform. Of course, not all MOsH were pro-active or forward-looking, and caution needs to be exercised about extrapolating too widely from a handful of examples. Further work on the activities of MOsH outside of London is needed. However, there seemed to be both a recognition of the changing nature of public health problems and an awareness of the need for a new kind of response to meet these.

A new approach to public health problems also required a different relationship between public health services and the public. Here too, we can find signs of a novel dynamic in the making. Early exhibitions tended to adopt a paternalistic and didactic approach, simply telling the public what to do and expecting them to follow suit. Some MOsH viewed the public as ignorant or apathetic, or felt that they were interested in the ‘wrong’ things, such as the quality of housing or litter. The moralistic and surveillance orientated aspects of interwar public health services had not disappeared entirely either. The focus on food hygiene in exhibitions during the 1950s, and later, safety in the home, could be seen as part of an attempt by public health practitioners to reach further into the working-class home, but there were other shifts on display too. MOsH were keen to speak to a wider section of the public than ever before. Specific groups, such as women and children, the working classes and ethnic minorities did attract special attention, but there was also a desire to reach the ‘general’ public. This was important, because the new conditions facing public health were to be found in the whole population and not just women or the working classes. As public health practitioners adjusted to deal with the changed pattern of disease and its aetiology, so too did their conception of the public. The public was no longer (if it ever had been) an undifferentiated mass. As the linkages between individual behaviour and the risk of developing a chronic condition became more apparent, the identification of people in need of help became clearer. At the same time, MOsH were increasingly aware that to improve public health, they needed to persuade individuals to adopt more healthy ways of living. There was thus a transition away from pure didacticism and towards a set of more responsive approaches aimed at changing behaviour. Furthermore, some MOsH considered in detail why they should be carrying out health education work; they worried about whether it worked; and if it did not, some MOsH were aware that this was not just because of an apathetic or ignorant public, but spoke also to the effectiveness or otherwise of their approaches.

Garnering a sense of what the public made of such shifts, is, however, more difficult to uncover. Attendance figures at exhibitions would suggest that the public did go along to such events, sometimes in large numbers. Once there, it would seem as if they found aspects of exhibitions entertaining and possibly enlightening, but the extent to which the displays changed individual behaviour is impossible to judge. What does come through is the ability of some members of the public to use the exhibition and the interaction with public health officials that this afforded to make complaints and raise grievances. When treated with caution, such complaints can offer insight into a sub-set of public opinion. As in other areas of health service provision, formal and informal complaints were made rarely, but they probably represent the views of others who were unwilling or unable to make their voices heard.^119^ The capacity of the public to speak back to public health services (and the ways in which this is recorded and framed within the MOsH reports) was limited, but it does indicate that the public was not an entirely passive recipient of health education and public health services. What the public thought, how they responded and how they behaved were to become crucial in health education in subsequent decades, but even in the 1950s and early 1960s we can find signs of both a more responsive MOH and a more vocal public than might have been expected. In public health exhibitions what was on display was not just a set of objects and images, but the very nature of the changing relationship between public health authorities and the public.

## Footnotes

1.
Report of the Medical Officer of Health [hereafter MOH] for Ealing, 1949, 24–5. On the significance of Woman’s Hour in this period see Kristin Skoog, ‘Neither Worker nor Housewife but Citizen: BBC’s Woman’s Hour 1946–55’, *Women’s History Review*, 26, 6 (2017), 953–74 and online (2017), 1–22.

2.

S.P.W. Chave , ‘Producing an Exhibition: An Exercise in Public Health Teaching’, Health Education Journal, 17, 2 (1959), 94–97.

3.

F.St.D. Rowntree , ‘The Health Exhibition’, Health Education Journal, 17, 2 (1959), 109–118.

4.

Abdel R. Omran , ‘The Epidemiologic Transition: A Theory of the Epidemiology of Population Change’, The Milbank Quarterly, 83, 4 (2005), 731–757; David Armstrong, ‘Chronic Illness: A Revisionist Account’, *Sociology of Health & Illness*, 36, 1 (2014), 15–27; George Weisz and Jesse Olszynko-Gryn, ‘The Theory of Epidemiologic Transition: The Origins of a Citation Classic’, *Journal of the History of Medicine and Allied Sciences*, 65, 3 (2010), 287–326; Chris Gilleard and Paul Higgs, ‘Revisionist or Simply Wrong? A Response to Armstrong’s Article on Chronic Illness’, *Sociology of Health & Illness*, 36, 7 (2014), 1111–15; George Weisz, *Chronic Disease in the Twentieth Century: A History* (Baltimore, MD: Johns Hopkins University Press, 2014).16279965

5.

J.N. Morris , Uses of Epidemiology (Edinburgh & London: E&S Livingstone, 1957).

6.

Jane Lewis , What Price Community Medicine? The Philosophy, Practice and Politics of Public Health since 1919 (Brighton: Wheatsheaf, 1986); Jane Lewis, ‘The public’s health: philosophy and practice in Britain in the twentieth century’, in *A History of Education in Public Health: Health That Mocks the Doctors’ Rules,* ed. by Elizabeth Fee, and R.M. Acheson (Oxford: Oxford University Press, 1991), 195–229; Charles Webster, *The Health Services since the War*,1: *Problems of Health Care, the National Health Service before 1957* (London: HMSO, 1988).

7.

Martin Gorsky , ‘Local Leadership in Public Health: The Role of the Medical Officer of Health in Britain, 1872–1974’, Journal of Epidemiology and Community Health, 61, 6 (2007), 468–472; John Welshman, ‘The Medical Officer of Health in England and Wales, 1900–74: Watchdog or Lapdog?’, *Journal of Public Health Medicine*, 19, 4 (1997), 443–50; Lesley Diack and David F. Smith, ‘Professional Strategies of Medical Officers of Health in the Post-War Period, 1: “Innovative Traditionalism”: The Case of Dr Ian MacQueen, MOH for Aberdeen 1952–1974, a “Bull-Dog” with the “Hide of a Rhinoceros” ’, *Journal of Public Health Medicine*, 24, 2 (2002), 123–9.1214158110.1093/pubmed/24.2.123

8.

John Pickstone , ‘Production, community and consumption: the political economy of twentieth-century medicine’, in Roger Cooter and John Pickstone (eds), Medicine in the Twentieth Century (Amsterdam: Harwood, 2000), 1–19.

9.
T.H. Marshall, ‘Citizenship and social class’, in *Citizenship and Social Class* (London: Pluto, 1992).

10.

Dorothy Porter , Health Citizenship: Essays in Social Medicine and Biomedical Politics (Berkeley & Los Angeles, CA: University of California Press, 2011).

11.

Dorothy Porter , ‘From Social Structure to Social Behaviour in Britain after the Second World War’, Contemporary British History, 16, 3 (2002), 58–80.

12.

Mike Savage , Identities and Social Change in Britain since 1940: The Politics of Method (Oxford: Oxford University Press, 2010); David Armstrong, *Political Anatomy of the Body: Medical Knowledge in Britain in the Twentieth Century* (Cambridge: Cambridge University Press, 1983).

13.

Helen McCarthy , ‘Social Science and Married Women’s Employment in Post-War Britain’, Past & Present, 233, 1 (2016), 269–305; Jon Lawrence, ‘Social-Science Encounters and the Negotiation of Difference in Early 1960s England’, *History Workshop Journal*, 77, 1 (2014), 215–39; Jon Lawrence, ‘Class, “Affluence” and the Study of Everyday Life in Britain, *c*1930–64’, *Cultural and Social History*, 10, 2 (2013), 273–99; Laura DuMond Beers, ‘Whose Opinion? Changing Attitudes Towards Opinion Polling in British Politics, 1937–64’, *Twentieth Century British History*, 17, 2 (2006), 177–205.

14.

John Coggon , What Makes Health Public? A Critical Evaluation of Moral, Legal and Political Claims in Public Health (Cambridge: Cambridge University Press, 2011).

15.

David Cantor , ‘Representing “the Public”: medicine, charity and the public sphere in twentieth-century Britain’, in Steve Sturdy (ed.), Medicine, Health and the Public Sphere in Britain: 1600–2000 (London: Routledge, 2002), 145–168, 160.

16.
See http://wellcomelibrary.org/moh/accessed 13.02.17.

17.

Sally Shuttleworth and Sally Frampton , ‘Constructing Scientific Communities: Citizen Science’, The Lancet, 385.9987 (2015), 2568.10.1016/S0140-6736(15)61150-326122151

18.

Megan Vaughan , ‘Healing and Curing: Issues in the Social History and Anthropology of Medicine in Africa’, Social History of Medicine, 7, 2 (1994), 283–295.1163933010.1093/shm/7.2.283

19.
Lewis, *op. cit.* (note 6).

20.

Charles Webster , ‘Health, Welfare and Unemployment during the Depression’, Past & Present, (1985), 204–230 Charles Webster, ‘Healthy or Hungry Thirties?’, *History Workshop Journal*, 13, 1 (1982), 110–29.1161790510.1093/past/109.1.204

21.
Webster, *op. cit.* (note 6), 377–8.

22.
Gorsky, *op. cit.* (note 7).

23.

Martin Gorsky , ‘Public Health in Interwar England and Wales: Did It Fail?’, Dynamis, 28 (2008), 175–198.1923033910.4321/s0211-95362008000100008PMC2647660

24.
Welshman, *op. cit.* (note 7).

25.
Diack and Smith, *op. cit.* (note 7).

26.

Hilary Marland , Health and Girlhood in Britain, 1874–1920 (Basingstoke: Palgrave Macmillan, 2013).

27.

Vicky Long and Hilary Marland , ‘From Danger and Motherhood to Health and Beauty: Health Advice for the Factory Girl in Early Twentieth-Century Britain’, Twentieth Century British History, 20, 4 (2009), 454–481.2048106110.1093/tcbh/hwp027PMC4513071

28.
Lewis, ‘The public’s health’, *op. cit.* (note 6).

29.

John Welshman , ‘“Bringing Beauty and Brightness to the Back Streets”: Health Education and Public Health in England and Wales, 1890–1940’, Health Education Journal, 56, 2 (1997), 199–209.

30.
Max Blythe, ‘A History of the Central Council for Health Education, 1927–68’ (unpublished DPhil, University of Oxford, 1987).

31.

Roger Davidson , Dangerous Liaisons: A Social History of Venereal Disease in Twentieth-Century Scotland (Amsterdam: Rodopi, 2000); Gareth Millward, ‘ “A Matter of Commonsense”: The Coventry Poliomyelitis Epidemic 1957 and the British Public’, *Contemporary British History*, online (2016), 1–23.

32.

Virginia Berridge , Marketing Health: Smoking and the Discourse of Public Health in Britain, 1945–2000 (Oxford: Oxford University Press, 2007), 71–75, 188–9.

33.
Ministry of Health, *Health Education: Report of a Joint Committee of the Central and Scottish Health Services Councils* (London: HMSO, 1964); Ian Sutherland, *Health Education – Half a Policy, 1968–86: Rise and Fall of the Health Education Council* (Cambridge: NEC Publications, 1987).

34.

Virginia Berridge and Kelly Loughlin , ‘Smoking and the New Health Education in Britain 1950s–1970s’, American Journal of Public Health, 95, 6 (2005), 956–964; Alex Mold, ‘ “Everybody Likes a Drink. Nobody Likes a Drunk”: Alcohol, Health Education and the Public in 1970s Britain’, *Social History of Medicine*, 30, 1 (2017), 612–36.15914816

35.

David Serlin , ‘Introduction: toward a visual culture of public health: from broadside to YouTube’, in Imagining Illness: Public Health and Visual Culture (Minneapolis, MN: University of Minnesota Press, 2010), xi–xxxvii; William H Helfand, ‘ “Some one sole unique advertisement”: public health posters in the twentieth century’, in *Imagining Illness: Public Health and Visual Culture* (Minneapolis, MN: University of Minnesota Press, 2010), 126–42; Tim Boon, ‘Health education films in Britain, 1919–39: production, genres and audiences’, in Graeme Harper and Andrew Moor (eds), *Signs of Life: Cinema and Medicine* (London: Wallflower Press, 2005), 45–57.

36.

Samuel J.M.M. Alberti , Morbid Curiosities: Medical Museums in Nineteenth-Century Britain (Oxford: Oxford University Press, 2011); Michael Sappol, ‘ “Morbid Curiosity”: The Decline and Fall of the Popular Anatomical Museum’, *Common-Place*, 4.2 (2004) http://www.common-place.org/vol-04/no-02/sappol/ (accessed 14 April 2015).

37.

Claudia Stein , ‘Organising the History of Hygiene at the Internationale Hygiene-Ausstellung in Dresden in 1911’, NTM Zeitschrift für Geschichte der Wissenschaften, Technik und Medizin, 21, 4 (2014), 355–387; Claudia Agostoni, ‘Popular Health Education and Propaganda in Times of Peace and War in Mexico City, 1890s–1920s’, *American Journal of Public Health*, 96.1 (2006), 52–61; Annmarie Adams, ‘The healthy Victorian city: the old London street at the international health exhibition of 1884’, in Zeynep Çelik, Diane Favro, and Richard Ingersoll (eds), *Streets: Critical Perspectives on Public Space* (Berkeley and Los Angeles, CA: University of California Press, 1996), 203–12.

38.

Julie K. Brown , Health and Medicine on Display: International Expositions in the United States, 1876–1904 (Cambridge, MA: MIT Press, 2009).

39.

Becky E. Conekin , The Autobiography of a Nation: The 1951 Festival of Britain (Manchester and New York: Manchester University Press, 2003); Sophie Forgan, ‘Festivals of Science and the Two Cultures: Science, Design and Display in the Festival of Britain, 1951’, *The British Journal for the History of Science*, 31 (1998), 217–40; Harriet Atkinson, *The Festival of Britain: A Land and Its People* (London: I.B. Tauris, 2012).

40.

Fife Clark , The Central Office of Information (London: George Allen & Unwin, 1970), 84.

41.
Due to differences between the MOsH jurisdictions and borough changes, the actual coverage is likely to be higher.

42.
Occupation statistics taken from the 1951 Census. See http://www.visionofbritain.org.uk/census/table_page.jsp?tab_id=EW1951COU_M27&u_id=10041790&show=&min_c=1&max_c=5Accessed 13.03.17.

43.

Anne Hardy , Salmonella Infections, Networks of Knowledge, and Public Health in Britain, 1880–1975 (Oxford: Oxford University Press, 2014).

44.

Matthew Smallman-Raynor and Andrew Cliff , Atlas of Epidemic Britain: A Twentieth-Century Picture (Oxford: Oxford University Press, 2012), 148–149.

45.

Andrew Rosen , The Transformation of British Life, 1950–2000: A Social History (Manchester: Manchester University Press, 2003), 14.

46.
Report of the MOH for Erith, 1949, 39.

47.
Report of the MOH for Wanstead and Woodford, 1955, 25.

48.
Report of the MOH for West Ham, 1951, 45.

49.

Mark Jackson , ‘Cleansing the air and promoting health: the politics of pollution in postwar Britain’, in Medicine, the Market and the Mass Media (Abingdon: Routledge, 2005), 221–243.; Peter Thorsheim, *Inventing Pollution: Coal, Smoke, and Culture in Britain since 1800*, (Ohio University Press, 2009); Christine L. Corton, *London Fog: The Biography* (Cambridge, MA: Harvard University Press, 2015).

50.
Report of the MOH for Woolwich, 1951, 27.

51.
Report of the MOH for London County Council, 1961.

52.
Report of the MOH for Leyton, 1957, 18.

53.
Welshman, *op. cit.* (note 29); Jane Lewis, *The Politics of Motherhood: Child and Maternal Welfare in England, 1900–39* (London: Croom Helm, 1980); Celia Davies, ‘The Health Visitor as Mother’s Friend: A Woman’s Place in Public Health, 1900–14’, *Social History of Medicine*, 1, 1 (1988), 39–59.

54.
Report of the MOH for London County Council, 1949, 79.

55.

Jane Seymour , ‘Not rights but reciprocal responsibility: the rhetoric of state health provision in early twentieth-century Britain’, in Assembling Health Rights in Global Context: Genealogies and Anthropologies (Abingdon: Routledge, 2013), 23–41.

56.
Marshall, *op. cit.* (note 9).

57.
Report of the MOH for East Ham, 1949, 31.

58.
See, for example, Report of MOH for Redbridge, 1969/ Hillingdon, 1969/ Brent, 1968/ Kingston upon Thames, 1971.

59.
Porter, *op. cit.* (note 10), 1–5, 204–16.

60.

Virginia Berridge , ‘Medicine and the Public: The 1962 Report of the Royal College of Physicians and the New Public Health’, Bulletin of the History of Medicine, 81, 1 (2007), 286–311.1736967210.1353/bhm.2007.0000PMC1894742

61.
Berridge, *op. cit.* (note 32), 250–1.

62.
Report of the MOH for Croydon, 1957, 42.

63.
Report of the MOH for Camberwell, 1959, 13–14.

64.
Morris, *op. cit.* (note 5), 19–23, 35–9.

65.

William G. Rothstein , Public Health and the Risk Factor: A History of an Uneven Medical Revolution (Rochester, NY: University of Rochester Press, 2003); Robert Aronowitz, ‘The social construction of coronary heart disease risk factors’, in *Making Sense of Illness: Science, Society and Disease* (Cambridge: Cambridge University Press, 1988), 111–44.

66.
Report of the MOH for Croydon, 1958, 49.

67.

P.K. Hendy , ‘Planning an Exhibition’, Health Education Journal, 17, 2 (1959), 98–104, 98.

68.
Hendy, *op. cit.* (note 67); Report of the MOH for Ilford, 1948/ Islington, 1956/Fulham, 1950/Stepney, 1950.

69.
Report of the MOH for Battersea, 1951/ Leyton, 1957/Wanstead and Woodford, 1955.

70.
Report of the MOH for Leyton, 1957, 16–22.

71.
Report of the MOH for Camberwell, 1958.

72.
Report of the MOH for Stepney, 1950, 60.

73.
Report of the MOH for Hayes, 1953, 20.

74.
Report of the MOH for Wembley, 1951, 26.

75.
Report of MOH for Enfield, 1956, 5.

76.
Report of the MOH for Leyton, 1950/Walthamstow, 1953/Camberwell, 1959.

77.
Report of the MOH for Edmonton, 1950/Battersea, 1951/Islington, 1954/Barking, 1959/Holborn, 1962.

78.
Report of the MOH for Edmonton, 1949, 4–5.

79.
Report of the MOH for Southgate, 1949, 35.

80.
Report of the MOH for Bexley, 1952, 6.

81.
Report of the MOH for Croydon, 1957, 42.

82.
Report of the MOH for Croydon, 1958, 48, 50.

83.
Report of the MOH for Greenwich, 1965, 264.

84.
Report of the MOH for Redbridge, 1966, 49.

85.
Report of the MOH for Bexley, 1952, 6.

86.

Gareth Millward , Vaccinating Britain: Mass Vaccination and the British Public since the Second World War (Manchester: Manchester University Press) (forthcoming).31487128

87.

*Report of the MOH for Camberwell*, 1958, 22.

88.
Rowntree, *op. cit.* (note 3), 109.

89.
Report of the MOH for Stepney, 1950, 60.

90.
Report of MOH for Camberwell, 1959, 10.

91.
Report of the MOH for Camberwell, 1961, 22.

92.

Roberta E. Bivins , Contagious Communities: Medicine, Migration, and the NHS in Post-War Britain (USA: Oxford University Press, 2015).

93.

Helen Pearson , The Life Project: The Extraordinary Story of Our Ordinary Lives (UK: Penguin Random House, 2016).

94.

Nancy Krieger , ‘Who and What Is a “Population”? Historical Debates, Current Controversies, and Implications for Understanding “Population Health” and Rectifying Health Inequities’, Milbank Quarterly, 90, 4 (2012), 634–681.2321642610.1111/j.1468-0009.2012.00678.xPMC3530737

95.
Morris, *op. cit.* (note 5), 38.

96.
Report of the MOH for Battersea, 1951, 52.

97.
Report of the MOH for Camberwell, 1961, 22.

98.
Report of the MOH for Acton, 1950, 32.

99.
Report of the MOH for Hornsey, 1953, 55.

100.
Report of the MOH for Woolwich, 1951, 26.

101.
There is a large literature on the history of disgust. A useful overview is provided by Richard Firth-Godbehere in ‘Ugh! A brief history of disgust’ https://emotionsblog.history.qmul.ac.uk/2012/02/ugh-a-brief-history-of-disgust/accessed 20.12.16.

102.
Report of the MOH for Hornsey, 1953, 55.

103.
Report of the MOH for Camberwell, 1959, 13–14.

104.
Report of the MOH for Stepney, 1950, 64.

105.

*Ibid.*, 63.

106.
Report of the MOH for Ealing, 1961, 21.

107.
Report of the MOH for Hampstead Borough, 1962, 33.

108.
Report of the MOH for Barking, 1952, 18.

109.
Report of the MOH for Wood Green, 1955, 5.

110.
Berridge, *op. cit.* (note 32), 203.

111.
Report of the MOH for Wembley, 1951, 26.

112.
Report of the MOH for Ealing, 1961, 20.

113.
Report of the MOH for Friern Barnet, 1959, 49.

114.
Report of the MOH for Camberwell, 1959, 14–15.

115.
Ministry of Health, *op. cit.* (note 33), 66–7.

116.
Statistics taken from http://www.barb.co.uk/resources/tv-ownership/and http://www.mediastudentsbook.com/content/case-study-outline-history-tv-uk accessed 13.02.17.

117.
Berridge and Loughlin, *op. cit.* (note 34).

118.
Ministry of Health, *op. cit.* (note 33), 9.

119.

Jonathan Reinarz and Rebecca Wynter (eds), Complaints, Controversies and Grievances in Medicine: Historical and Social Science Perspectives (London: Routledge, 2015).

